# Preparation and Performance Evaluation of Ionic Liquid Copolymer Shale Inhibitor for Drilling Fluid Gel System

**DOI:** 10.3390/gels10020096

**Published:** 2024-01-26

**Authors:** Zhiwen Dai, Jinsheng Sun, Zhuoyang Xiu, Xianbin Huang, Kaihe Lv, Jingping Liu, Yuanwei Sun, Xiaodong Dong

**Affiliations:** 1 Key Laboratory of Unconventional Oil & Gas Development, China University of Petroleum (East China), Ministry of Education, Qingdao 266580, China; 2 School of Petroleum Engineering, China University of Petroleum (East China), Qingdao 266580, China; 3 CNPC Engineering Technology R & D Company Limited, Beijing 102206, China

**Keywords:** shale gas, ionic liquid copolymer, shale inhibitor, water-based drilling fluid gel system

## Abstract

An inhibitor that can effectively inhibit shale hydration is necessary for the safe and efficient development of shale gas. In this study, a novel ionic liquid copolymer shale inhibitor (PIL) was prepared by polymerizing the ionic liquid monomers 1-vinyl-3-aminopropylimidazolium bromide, acrylamide, and methacryloyloxyethyl trimethyl ammonium chloride. The chemical structure was characterized using fourier transform infrared spectroscopy (FT-IR) and hydrogen-nuclear magnetic resonance (H-NMR), and the inhibition performance was evaluated using the inhibition of slurrying test, bentonite flocculation test, linear expansion test, and rolling recovery test. The experimental results showed that bentonite had a linear expansion of 27.9% in 1 wt% PIL solution, 18% lower than that in the polyether amine inhibitor. The recovery rate of shale in 1 wt% PIL was 87.4%. The ionic liquid copolymer could work synergistically with the filtrate reducer, reducing filtration loss to 7.2 mL with the addition of 1%. Mechanism analysis showed that PIL adsorbed negatively charged clay particles through cationic groups, which reduced the electrostatic repulsion between particles. Thus, the stability of the bentonite gel systems was destroyed, and the hydration dispersion and expansion of bentonite were inhibited. PIL formed a hydrophobic film on the surface of clay and prevented water from entering into the interlayer of clay. In addition, PIL lowered the surface tension of water, which prevented the water from intruding into the rock under the action of capillary force. These are also the reasons for the superior suppression performance of PIL.

## 1. Introduction

Shale gas formation has strong water sensitivity [1,2] and developes fractures [1,3]. The problem of wellbore collapse is still very serious when using the commonly used strong inhibition and strong plugging oil-based drilling fluid in the drilling process of long horizontal sections [4], and the risk of sticking is great [5]. Compared with synthetic-based and oil-based drilling fluids, water-based drilling fluid gel systems have the advantages of having low cost and highly environmentally friendly performance in the process of drilling construction, and they are internationally recognized as the development direction of shale gas development technology [6]. However, due to the hydration characteristics of shale, the wellbore instability of shale formation drilled with water-based drilling fluid is still a major technical problem [7]. In addition, as the operation time increases, the continued interaction of shale and water can further cause enlargement of the borehole and even well-wall collapse.

The inhibitors in water-based drilling fluids mainly refer to shale inhibitors and hydrate inhibitors [8,9]. The main function of shale inhibitors is to inhibit clay hydration and stabilize the wellbore. At present, it is difficult to solve the problem of wellbore collapse in long-horizontal-section drilling using the methods of overcoming shale water sensitivity and plugging fractures [10]. The reason is that there is a lack of shale inhibitors that can effectively inhibit shale hydration. High-concentration potassium ions can effectively inhibit shale expansion [11], and amine inhibitors have a good inhibition effect on mudstone [12], but their inhibitive performance is insufficient to solve the problem of the hydration of shale gas formation [13]. Therefore, it is urgent to develop inhibitors to meet the needs of exploration and development of shale gas [14,15].

Ionic liquids are liquids composed entirely of ions [16]. They are highly solvated non-coordination media. A variety of organic and inorganic substances can be dissolved in them [17]. These properties have led to extensive research in various fields, such as biomass conversion [18], heat storage, lubrication [19], gas separation, electrolytes [20], water treatment, EOR [21], soil remediation, and material synthesis. In recent years, ionic liquids with unique properties have been applied to water-based drilling fluids as shale inhibitors and have shown good inhibition performance and environmental protection [22,23].

The most commonly used shale inhibitors are imidazole ionic liquids and ammonium ionic liquids, both of which show better inhibition performance at lower concentrations, and imidazole ionic liquids have good thermal stability [23,24]. Jia H. et al. [25] evaluated the inhibition performance of Gemini surface activity ionic liquids. They found that two charged head groups and two hydrophobic tail groups were able to enhance the adsorption, intercalation, and hydrophobic modification of ionic liquids, resulting in excellent inhibition properties. Rahman M. T. et al. [23] found that tetramethylammonium chloride inhibited clay swelling better than 1-ethyl-3-methylimidazolium chloride, and extending the ionic liquid chain segment can enhance the inhibitory effect. Short-chain alkyl substituents are beneficial for improving inhibition performance [26,27]. The more there are, the better their hydrophobic effect, thereby preventing water from entering the interlayer domain and reducing hydration. Compared to ionic liquids, ionic liquid copolymers can simultaneously have more cationic groups [28], longer chain segments, and more short-chain alkyl substituents, which make them a promising potential shale inhibitor [22]. However, fewer studies on the use of ionic liquid copolymers as inhibitors have been reported.

In this paper, an ionic liquid monomer 1-vinyl-3-aminopropyl imidazole bromide (VAPIMBr) was prepared. An ionic liquid copolymer (PIL) was prepared with VAPIMBr, acrylamide (AM), and methacryloyloxyethyl trimethyl ammonium chloride (DMC). PIL showed a good shale inhibition performance and good compatibility with drilling fluid, which had a good application prospect in shale gas drilling fluid.

## 2. Results and Discussion

### 2.1. Characterization of PIL

#### 2.1.1. Chemical Structure

**Figure 1:**
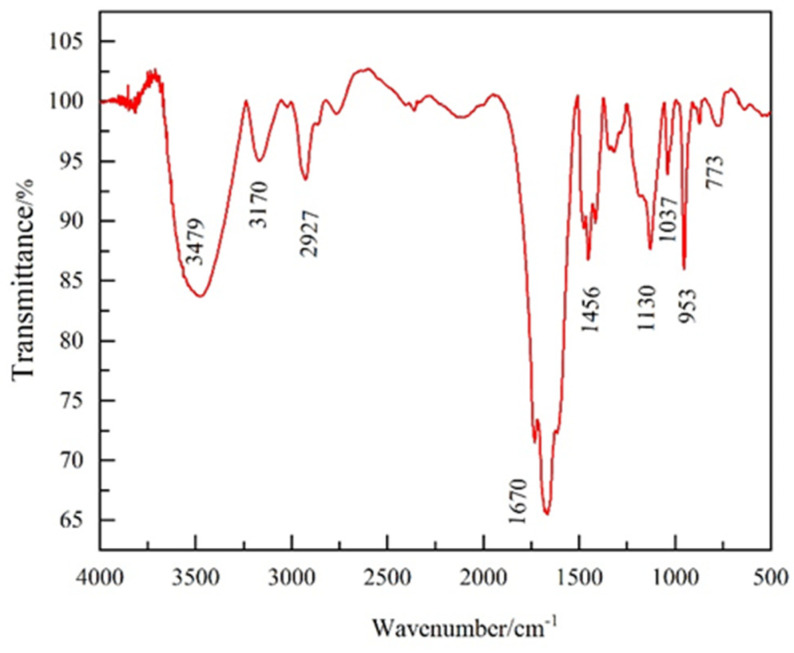
The FT-IR spectrum of PIL.

As shown in Figure 1, the broad peak of wavelength 3479 cm^−1^ is the stretching vibrational peak of the N-H bond, corresponding to the amide group. The peak at 3170 cm^−1^ is the stretching vibrational peak of C-H; 2927 cm^−1^ is the stretching vibrational peak of C-H, corresponding to the imidazolium ring substituent [29]; 1670 cm^−1^ and 1456 cm^−1^ are the backbone vibrational peaks of C=N and C=C, respectively; 773 cm^−1^ is the hydrocarbon planar out-of-plane oscillating bending vibrational peaks corresponding to the imidazole ring; 1037 cm^−1^ and 1456 cm^−1^ are methyl and methylene bending vibrational peaks corresponding to the methyl and methylene groups in DMC, respectively [30]. From the FT-IR analysis, it is clear that the synthesized PIL contains the characteristic functional groups of VAPIMBr, AM, and DMC, indicating that the synthesis is successful.

**Figure 2:**
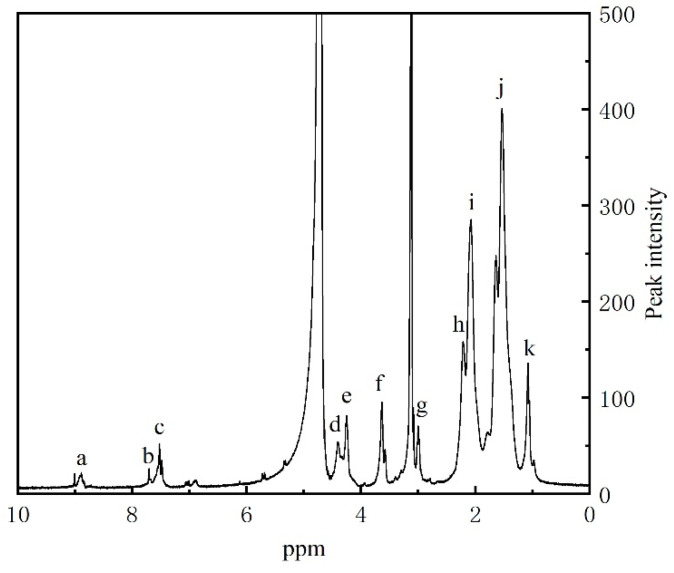
The H-NMR spectrum of PIL.

From Figure 2, it can be seen that peak a at 8.9 ppm corresponds to the imidazole tip. The single peak b at 7.74 ppm and peak c at 7.52 ppm correspond to the proton hydrogens at positions 4 and 5 of the imidazole ring, respectively. The peak d at 4.4 ppm corresponds to the hydrogen of aminopropyl-CH_2_. The peak e at 4.25 ppm and the peak f at 3.67 ppm correspond to the hydrogen in the methylene and methyl groups of DMC. The peak g at 3.03 ppm corresponds to the hydrogen of N-CH_3_ on DMC. The peak h at 2.34 ppm, peak i at 2.08 ppm, and peak j at 1.53 ppm correspond to the hydrogen on the carbon skeleton. The peak k at 1.08 ppm corresponds to the hydrogen of C-CH_3_ on DMC. Thus, H-NMR indicates that PIL has a designed analytical structure.

#### 2.1.2. Thermal Stability

**Figure 3:**
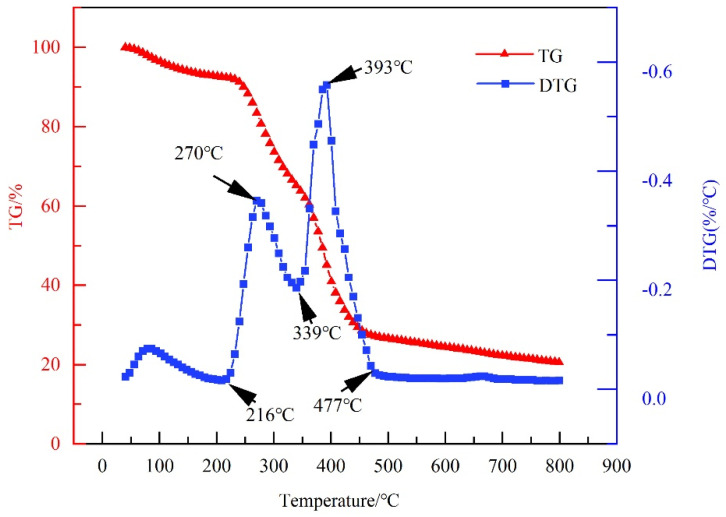
Thermogravimetric analysis of PIL.

It can be seen from Figure 3 that a small amount of weight loss occurred before 155 °C, which was mainly caused by the volatilization of a small amount of water molecules in the sample. The subsequent thermal decomposition process was divided into three stages. The first stage was 155 °C–216 °C, in which the bound water of the amide group in the PIL molecular structure volatilized [31]. The second stage was from 216 °C to 477 °C, when a large amount of thermal weight loss occurred. The rapid decomposition of the polymer between 216 °C and 339 °C was caused by the decomposition of the polymer branched chains commencing. From 339 °C to 477 °C, the main chain of PIL was decomposed. In the third stage, the remaining substances in the polymer sample were continuously carbonized and decomposed after 477 °C.

### 2.2. Inhibition Performance of PIL

**Figure 4:**
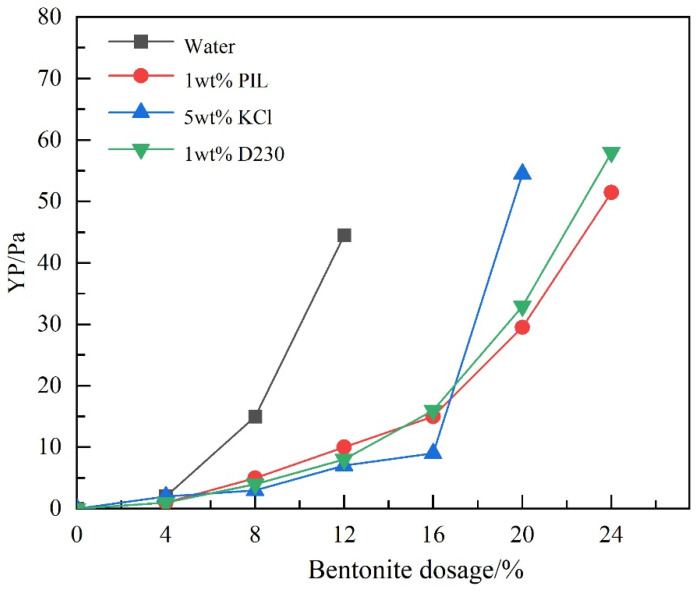
Variation of the yield point (YP) of different solutions with dosage of bentonite.

The slower the increase rate of the yield point, the stronger the ability to inhibit the hydration and dispersion of bentonite. Figure 4 shows that the yield point of deionized water increased significantly when the amount of bentonite added exceeded 8 wt% because bentonite tended to hydrate and disperse and form a “house card” structure in solution. When the bentonite dosage rose to 16 wt%, the yield point of the 1 wt% PIL solution, 5 wt% potassium chloride (KCl) solution, and 1 wt% polyether amine (D230) solution was less than that of deionized water. And the yield point of the KCl solution was the smallest, since there were more exchangeable cations. When the amount of bentonite added exceeded 16%, the yield points of the three solutions were rapidly increasing, but the yield points of PIL solution and D230 solution were much smaller than that of the KCl solution, indicating that the three inhibitors still had inhibition of bentonite hydration slurring ability. As the amount of bentonite continued to increase, the yield point of the PIL solution was smaller than that of the D230 solution, and the difference gradually increased.

**Figure 5:**
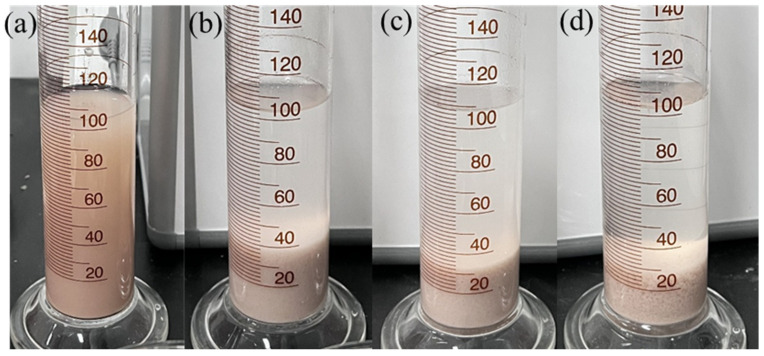
Results of bentonite flocculation experiment with different concentrations of PIL after 10 h: (**a**) deionized water, (**b**) 0.5 wt%, (**c**) 1 wt%, (**d**) 2 wt%.

Bentonite particles were dispersed into a stable colloidal system in deionized water due to hydration. The inhibitor in the water would affect the dispersion stability of bentonite particles. The height of bentonite flocculation in different concentrations of PIL solutions is shown in Figure 5. The dispersion without the addition of PIL showed no significant sedimentation stratification (Figure 5a). Bentonite particles in different concentrations of PIL showed obvious sedimentation stratification and different degrees of flocculation. The higher the concentration of PIL in the dispersion, the smaller the flocculation height and the larger the bentonite particles. When the concentration of PIL was 1 wt%, the settlement level of bentonite flocculation height was 32 mL. This was due to the fact that PIL adsorbed onto the negatively charged bentonite surface through electrostatic action, inhibiting further hydration and dispersion of the bentonite particles and increasing the chance of inter-particle aggregation to a larger size.

**Figure 6:**
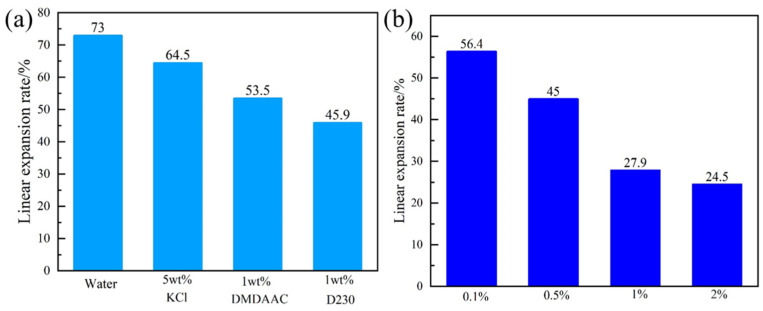
Linear expansion rate of artificial bentonite columns for 20 h: (**a**) in different inhibitor solutions, (**b**) in different concentrations of PIL solution.

The performance of PIL to inhibit the bentonite hydration expansion was comparatively analyzed with KCL, diallyl dimethylammonium chloride (DMDAAC), and D230 using linear expansion experiments. Figure 6a shows that the linear expansion rate of bentonite columns immersed in water was 73%, while the expansion rates in 5 wt% KCl solution, 1 wt% DMDAAC solution, and 1 wt% D230 solution were 64.5%, 53.5%, and 45.9%, respectively. It showed that the conventional shale inhibitors KCl, DMDAAC, and D230 could inhibit the expansion of shale hydration, but the effect was not good. Figure 6b shows that 0.5 wt%, 1 wt%, and 2 wt% of PIL reduced the expansion rate to 45.1%, 27.9%, and 24.5%, respectively. Different concentrations of PIL showed better inhibition performance of clay hydration expansion than DMDAAC. When the concentration of PIL was 0.5 wt%, the inhibition performance was equivalent to that of 1 wt% D230, which may be because the molecular chain of PIL had a large number of positively charged groups. With the increase of PIL concentration, the inhibition performance of clay hydration expansion was better.

**Figure 7:**
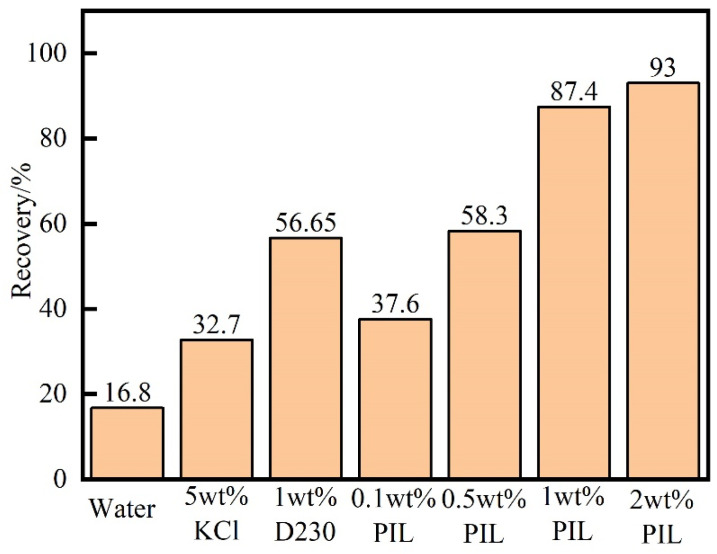
Recovery rate of shale fragments in different inhibitor solutions (aging at 120 °C for 16 h).

As shown in Figure 7, the recovery rate of shale in water was 16.8%, which indicated that the shale fragments had pretty good hydration dispersing ability. In 5 wt% KCl solution and 1 wt% D230 solution, the rolling recovery rate increased to 32.7% and 56.65%, respectively. KCL had poor inhibition ability to shale, which was easy to hydrate and disperse, and the D230 was significantly better than KCL. When the concentration of PIL solution was 1 wt%, the recovery rate of shale reached 87.4%, which was 70.6% higher than that in water. PIL significantly improved the recovery rate of shale, and its performance was superior to the commonly used shale inhibitors KCl and D230. In addition, as the concentration of PIL in the solution increased, the recovery rate of shale also gradually improved. PIL formed an adsorbent film on shale fragments, preventing the disintegration of shale fragments during the rolling process.

### 2.3. Analysis of PIL Inhibition Mechanism

**Figure 8:**
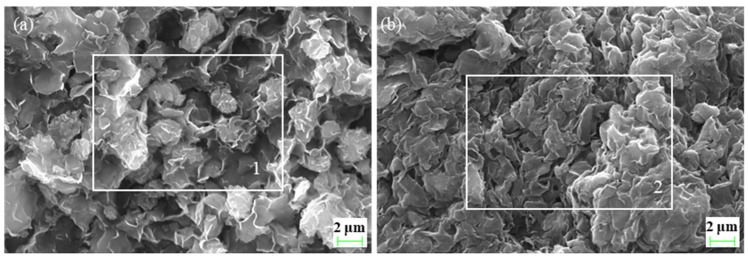
SEM images of bentonite: (**a**) bentonite slurry, white frame selection region 1 refers to the region for element analysis; (**b**) bentonite slurry with 1 wt% PIL, white frame selection region 2 refers to the region for element analysis.

As shown in Figure 8a, the original bentonite was dispersed in flakes, with particle sizes ranging from a few hundred nanometres to dozens of micrometers, and the particle size distribution was uneven. After adding PIL to the bentonite slurry, the bentonite flakes were stacked and the particle size distribution became concentrated (Figure 8b). Table 1 shows the normalized mass ratios of the elemental analysis performed on the selected regions 1 and 2 in Figure 8, respectively. The bentonite surfaces of two regions were enriched in the oxygen, silicon, and aluminum elements, which was consistent with the theoretical chemical formula. The carbon content on the surface of the PIL-treated bentonite reached 17.78%, while the original bentonite was free of carbon. It indicated that PIL had been adsorbed onto bentonite.

**Figure 9:**
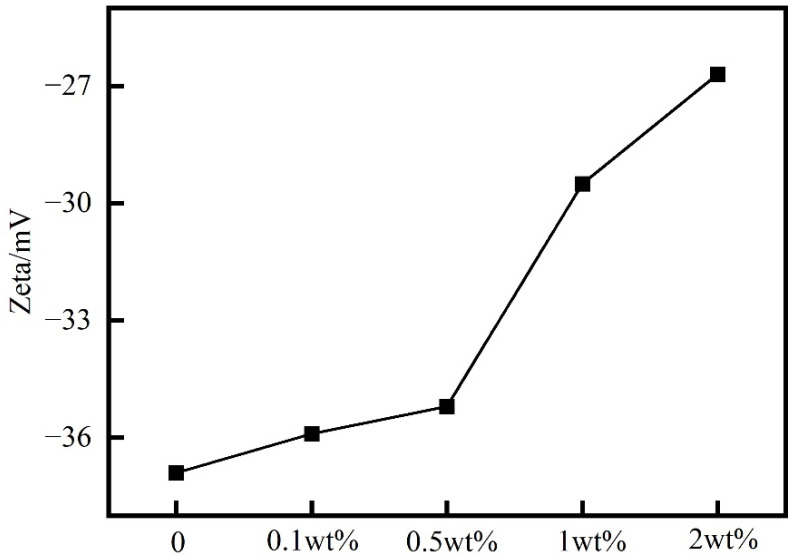
Zeta potential of 4% bentonite slurry with different concentrations of PIL solutions.

The bentonite slurry is a colloidal dispersion system formed by bentonite particles in deionized water, and the absolute value of the Zeta potential of the clay particles reflects the stability of the dispersion. After hydrolysis, PIL exhibits a positive charge due to the presence of cationic groups such as imidazole and ammonium on the side chains of the PIL molecule. Due to lattice substitution and other factors, bentonite carries a negative charge in water. As shown in Figure 9, the absolute value of the zeta potential decreased with increasing concentration of PIL in the bentonite slurry. This indicates that PIL could adsorb on the surface of bentonite, which is consistent with the SEM of bentonite results [22]. Colloidal systems with an absolute value of a zeta potential greater than 30 were considered stable. When the concentration was 2 wt%, the absolute value of the zeta potential still reached 27, indicating that PIL destabilized the dispersion of the prehydrated bentonite slurry, but to a lower extent [25].

**Figure 10:**
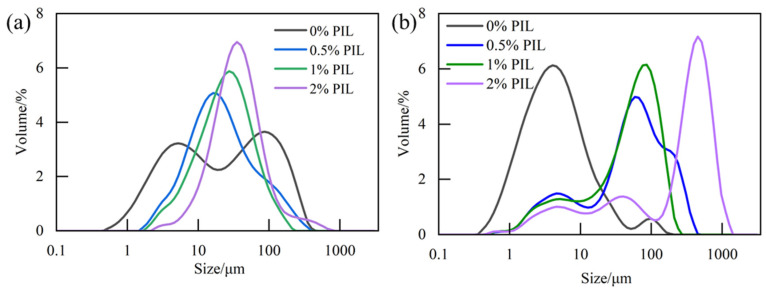
Particle size distribution of 4% bentonite slurry with different concentrations of PIL: (**a**) before aging, (**b**) after aging at 120 °C for 16 h.

Figure 10a shows that before aging, during the increase of PIL concentration from 0 wt% to 2 wt%, the particle size distribution curves of bentonite slurry generally showed a rightward shifting trend, and the particles were more monodisperse. After the clay particles adsorbed the inhibitor, the surface negative electronegativity was reduced, which lowered the repulsive force between the clay particles and to a certain extent destroyed the original dispersion stability of the clay particles. Some of the clay particles were agglomerated into large particles, which led to a right shift of the particle size distribution curve. After aging, the particle size distribution curve was still shifted to the right with the increase of PIL concentration. But, the range of the particle size distribution became larger because the high temperature promoted the interaction of PIL with bentonite particles and bentonite particles with bentonite particles [32].

**Figure 11:**
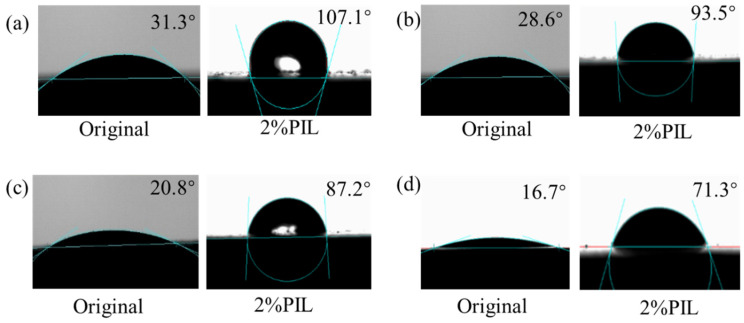
Water contact angle of shale pieces after aging at different temperatures: (**a**) 25 °C, (**b**) 80 °C, (**c**) 100 °C, (**d**) 120 °C.

Changes in surface wettability of shale pieces in 2 wt% PIL after aging at different temperatures were investigated. As shown in Figure 11, the original shale surface was initially hydrophilic at 25 °C, and the water contact angle of the original shale piece was 31.3°. As the temperature increased, the contact angle of the original shale piece decreased, i.e., it became more hydrophilic. This may be due to changes in surface roughness and the free energy of shale pieces, which were caused by the dissolution of soluble substances and increased interaction between shale and water due to temperature [33]. After aging in 2 wt% PIL solution, the water contact angle of the shale increased to 107.1° at room temperature, and the surface wettability changed from hydrophilic to hydrophobic. With the increase in temperature, the water contact angle of shale treated with PIL was gradually decreased, and the water contact angle was 71.3° at 120 °C, but it was still much larger than that of the original shale piece at 120 °C (16.7°). This suggests that PIL formed a hydrophobic film after adsorption on the surface of the original shale, preventing water from entering further into the bentonite particle crystals [25], which served to stabilize the well wall and inhibit the hydration of the formation clay.

**Figure 12:**
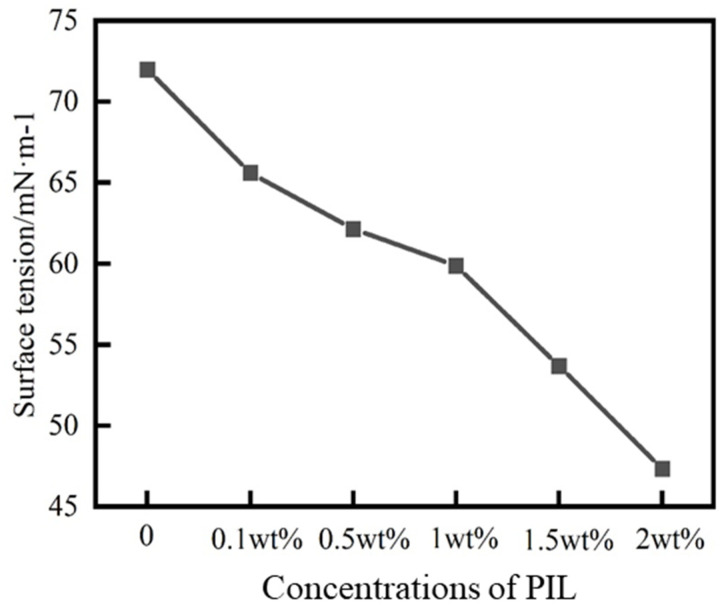
Surface tension of different concentrations of PIL solutions.

Figure 12 shows that with the increase of the PIL concentration, the surface tension of solution gradually decreased, and when the concentration was 2 wt%, the surface tension decreased from 72 mN/m to 47.35 mN/m. The decrease in surface tension reduced the capillary force between the drilling fluid filtration loss and the formation rock. The capillary force was generally considered to be the driving force for the drilling fluid filtration loss to enter the pore space of the formation [34]. Thus, PIL could attenuate water intrusion into the formation.

### 2.4. Compatibility with Drilling Fluid

**Figure 13:**
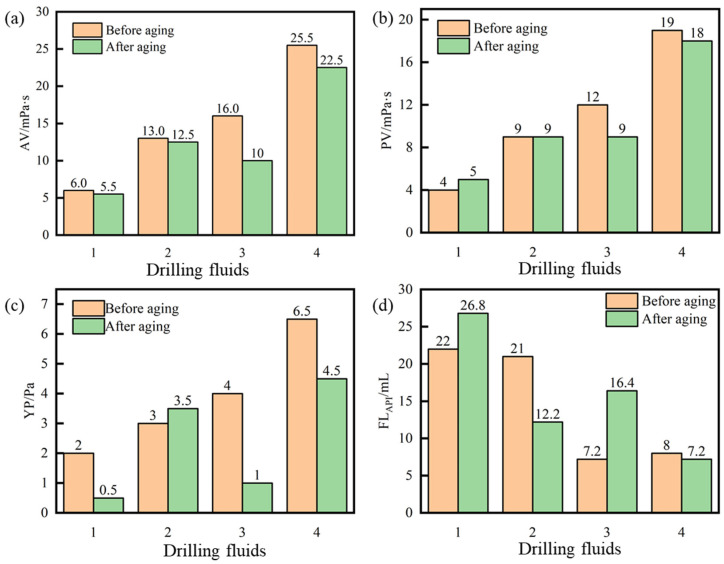
Rheological and filtration properties of drilling fluids before and after aging at 120 °C: (**a**) apparent viscosity (AV), (**b**) plastic viscosity (PV), (**c**) YP, (**d**) API filtration loss (FL_API_).

Figure 13 shows that the viscosity of the 4% bentonite slurry before and after aging was essentially unchanged, the yield point decreased, and the API filtration loss became larger. High temperatures can disrupt the dispersion stability of bentonite particles. The viscosity and yield point of bentonite slurry increased with the addition of PIL. The filtration loss of the bentonite slurry with 1 wt% PIL after aging at 120 °C decreased from 26.8 mL to 12.2 mL, indicating that PIL had a filtration reduction effect. After adding the filtration loss reducer PAC-LV, the viscosity of 4% bentonite slurry increased significantly and the filtration loss decreased. Compared with PIL, PAC-LV had a better filtration loss reduction performance. After adding both PAC-LV and PIL to the 4% bentonite slurry, the filtration loss could be reduced from 16.4 mL to 7.2 mL. The filtration reduction effect was better than adding the two additives alone, indicating that PIL could be synergized with PAC-LV.

## 3. Conclusions

In this work, we have developed a novel ionic liquid copolymer (PIL) and explored its potential application as a shale inhibitor for water-based drilling fluid gel systems. FT-IR and H-NMR tests showed that the synthesized PIL had a designed molecular structure. The inhibition evaluation experiments showed that PIL had better inhibition performance than the inhibitor D230 in preventing bentonite slurring. After adding bentonite to the PIL solution, the particles flocculated and precipitated. PIL had better inhibition performance for clay hydration and expansion than the traditional inhibitor KCl. For PIL, the cationic groups on the molecular chain can be easily adsorbed on the clay particles’ surface, which reduces the negative charge of the clay surface and compressed diffusion electric double layer of the clay particles. PIL formed a stable temperature-resistant hydrophobic adsorption film on the clay particles, preventing water from entering further into the interlayer of the clay particles. The surface activity of PIL reduced the surface tension of water, which impaired the driving force of water to enter the capillary pores of rock. In addition, PIL had good compatibility with bentonite slurry and filter loss reducers and could be synergized with PAC-LV. The present work inspired the development of novel ionic liquid copolymer inhibitors.

## 4. Materials and Methods

### 4.1. Materials

AM (99 wt%), DMC (75 wt% solutions), acetonitrile (99 wt%), diallyl dimethylammonium chloride (60 wt% solutions), potassium persulfate (99.5 wt%), sodium bisulfite (99.9 wt%), polyether amine (average Mn~230), and potassium chloride (99.5 wt%) were purchased from Shanghai Mclean Biochemical Technology Co., Ltd. (Shanghai, China) Bentonite was purchased from Huai’an County Tengfei bentonite Development Co., Ltd. (Huai’an, China) Polyanionic cellulose (PAC-LV) was purchased from Shandong Deshunyuan Petroleum Technology Co., Ltd. (Dongying, China) Shale cuttings and outcrops are from Sichuan, China. VAPIMBr was produced using the substitution reaction of 1-vinylimidazole and 3-bromopropylamine hydrobromide in acetonitrile.

### 4.2. Preparation of PIL

**Figure 14:**
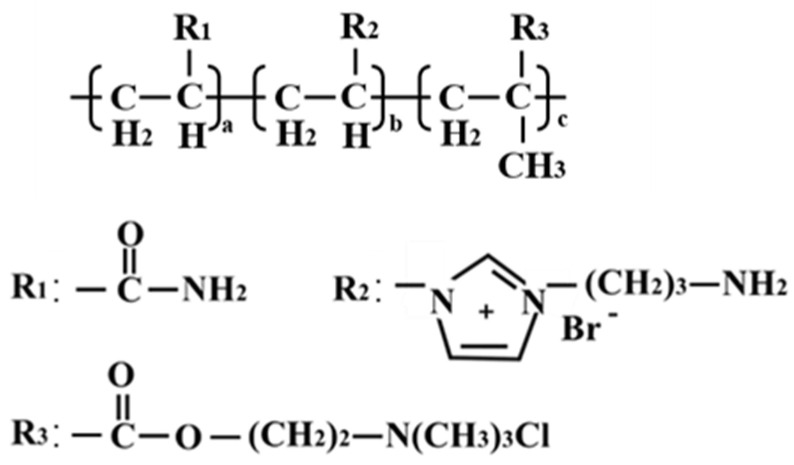
The molecular structure of PIL.

VAPIMBr, AM, and DMC were added into a flask containing water and stirred until fully dispersed. The mixture in the flask was heated to 55 °C and deoxidized with nitrogen for 30 min. Then, the initiator (potassium persulfate and sodium bisulfite) was added to the flask. The PIL was obtained after reaction for 7.5 h, washed with ethanol 2–3 times, and dried under 105 °C. The molecular structure of PIL is shown in Figure 14.

### 4.3. Characterization of PIL

#### 4.3.1. FT-IR Analysis

The infrared spectra of PIL were measured using an infrared spectrometer (Shimadzu irtracer-100, Kyoto, Japan). A small amount of each dried PIL samples were taken with tweezers and put into an agate mortar. An appropriate amount of KBr was mixed with PIL, and the mixture was ground into powder in one grinding direction. The powder was pressed at a pressure of 2 Ton for 5 min, and the prepared tablets were put into an infrared spectrometer to measure the FT-IR spectrum.

#### 4.3.2. H-NMR Spectra Analysis

Nuclear magnetic resonance hydrogen spectroscopy (H-NMR) can be used to determine the molecular structure of polymers. The H-NMR spectrum of PIL was studied using an NMR spectrometer (Bruker Ascend-400, Fällanden, Switzerland). Deuterated chloroform was used as the solvent.

#### 4.3.3. Thermogravimetric Analysis

The thermal stability of PIL was analyzed using a thermogravimetric analyzer (Mettler Toledo TGA2, Columbus, OH, USA). A small sample of dried PIL was placed in a zeroed crucible. The temperature was increased from 40 °C to 800 °C at a heating rate of 10 °C/min and then stopped. Nitrogen was used as a protective gas during heating.

### 4.4. Performance Evaluation of PIL

#### 4.4.1. Bentonite Slurrying Test

**Figure 15:**
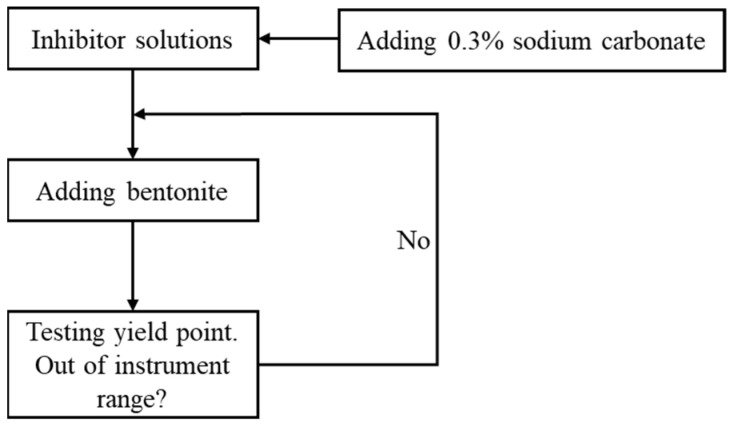
Flowchart of bentonite slurrying test.

During the drilling process, the intrusion of bentonite-containing drill cuttings into the drilling fluid system can lead to changes in the rheological properties of the drilling fluid. Therefore, the slurrying ability of bentonite can be inhibited by adding inhibitors to the water-based drilling fluid. After adding 0.3% sodium carbonate to the different inhibitor solutions, a certain mass of bentonite was added to the different inhibitor solutions. The dispersion was stirred for 20 min. Then, the yield point (YP) was tested using a six-speed rotational viscometer (Qingdao Tongchun Petroleum Instrument, Qingdao, China). When YP exceeded the measurement range of the instrument, the addition of bentonite to the solution was terminated. The flowchart is shown in Figure 15.

#### 4.4.2. Bentonite Flocculation Test

Inhibitors with positively charged groups may affect the hydration of bentonite. After adding 100 mL PIL solution with different concentrations to each measuring cylinder, 4 g of bentonite was added to each cylinder. Each cylinder was shaken to mix well, turned back and forth for 1 min, and then left to stand for 10 h. The level of bentonite flocculation height in the measuring cylinder was recorded.

#### 4.4.3. Linear Expansion Test

**Figure 16:**
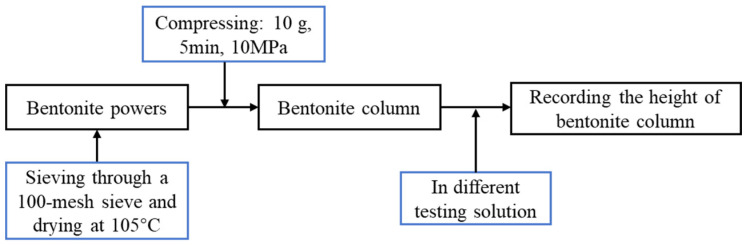
Flowchart of linear expansion test.

Bentonite was sieved through a 100-mesh sieve and dried at 105 °C. Then, an artificial bentonite column was prepared by adding 10 g of bentonite powder to the pressure cylinder at 10 MPa for 5 min [35]. The height of bentonite columns in different test solutions for 20 h was recorded using a dual-channel pycnometer expansion meter (Qingdao Tongchun Petroleum Instrument, Qingdao, China). The above method was adopted to evaluate the linear expansion of the bentonite column in deionized water, 0.1 wt% PIL, 0.5 wt% PIL, 1 wt% PIL, 2 wt% PIL, 5 wt% KCl, 1 wt% DMDAAC, and 1 wt% D230, respectively. The flowchart is shown in Figure 16.

#### 4.4.4. Rolling Recovery Test

**Figure 17:**
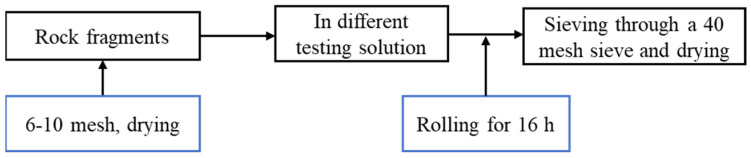
Flowchart of rolling recovery test.

In total, 6–10 mesh rock fragments were dried at 105 °C for 24 h. A total of 20 g of rock fragments were hot rolled under different test conditions in an aging tank for 16 h using a high-temperature roller heating furnace (Qingdao Tongchun Petroleum Instrument GW300-PLC, Qingdao, China). The hot-rolled rock fragments were sieved through a 40-mesh sieve. The remaining fragments on the sieve were dried in an oven at 105 °C for 24 h. Rolling recoveries of shale rock fragments were determined by aging in water, 0.1 wt%–2 wt% PIL, 5 wt% KCl, and 1 wt% D230, respectively. The flowchart is shown in Figure 17.

(1) $$\mathrm{Recovery}=\frac{M_{1}}{20}\;\times \;100\%$$

where *M*_1_ was the weight of the remaining dried shale rock fragments on the sieve.

### 4.5. Mechanism Analysis

#### 4.5.1. Scanning Electron Microscope (SEM) and Surface Elemental Analysis

Bentonite slurry was centrifuged, and the lower precipitate was taken and dried at 80 °C for 24 h. After sputtering a gold film on the precipitate, the apparent morphology was observed using scanning electron microscopy (Zeiss EVO-15LS, Oberkochen, Germany), and surface elemental analysis was performed using an energy dispersive spectrometer (EDS). The scanning voltage was 20 kV. The same test procedure was carried out with bentonite slurry with the addition of 1 wt% PIL.

#### 4.5.2. Zeta Potential Test

In total, 0.1 wt% PIL, 0.5 wt% PIL, 1 wt% PIL, 2 wt% PIL were added to 400 mL of 4.0 wt% bentonite slurry, respectively. Then, the zeta potential of bentonite slurry was measured using a potential instrument (Malvern Zetasizer Nano Z, Malvern, UK) after stirring for 20 min. The bentonite slurry was aged at 120 °C for 16 h, and the zeta potential was measured again.

#### 4.5.3. Particle Size Test

Different concentrations of PIL were added to the bentonite slurry. The particle size distribution of the bentonite slurries was measured before and after aging at 120 °C for 16 h using a laser diffraction method, using a particle size analyzer (Malvern Mastersizer 3000, Malvern, UK).

#### 4.5.4. Contact Angle Test

The shale was sliced, sanded, and polished. Then, the shale pieces were placed in 2 wt% PIL solution and hot rolled at room temperature (25 °C), 80 °C, 100 °C and 120 °C for 16 h, respectively, then removed and dried. The water contact angle of the treated shale pieces was measured using a contact angle meter (Dataphysics OCA-25, Filderstadt, Germany), after 5 s of stabilization.

#### 4.5.5. Surface Tension Test

PIL solutions with concentrations of 0.1 wt%, 0.5 wt%, 1 wt%, 1.5 wt%, and 2 wt% were prepared, respectively. The surface tension of the PIL solution was tested using the hanging plate method, using a dynamic contact angle tensiometer (Dataphysics DCAT21, Filderstadt, Germany).

### 4.6. Compatibility with Drilling Fluid

To investigate the compatibility of PIL with drilling fluid, drilling fluid was prepared using the following four formulas. The 4% bentonite slurry was prepared by adding 16 g of bentonite and 0.56 g of sodium carbonate into 400 mL of deionized water and aged at room temperature for 24 h. After aging, the drilling fluids of the four formulas were prepared by adding other additives and stirring for 20 min. Then, the rheological parameters and filtration loss were measured before and after aging at 120 °C to analyze the effect of PIL addition on the performance of drilling fluid.

No. 1: 4 wt% bentonite slurry

No. 2: 4 wt% bentonite slurry + 1 wt%PIL

No. 3: 4 wt% bentonite slurry + 0.5 wt% PAC-LV

No. 4: 4 wt% bentonite slurry + 0.5 wt%PAC-LV + 1 wt%PIL

## Figures and Tables

**Table 1:** Results of surface elemental analysis.

Element	Region 1	Region 2
Oxygen	42.70%	41.10%
Silicon	41.21%	26.99%
Aluminum	9.94%	7.40%
Calcium	2.82%	1.02%
Iron	2.56%	3.33%
Potassium	0.68%	1.89%
Chlorine	0.09%	0.39%
Magnesium	0.01%	0.11%
Carbon	0	17.78%

## Data Availability

All data and materials are available on request from the corresponding author. The data are not publicly available due to ongoing researches using a part of the data.
